# Allium leafminer (Diptera: Agromyzidae) host preference: implications for developing a trap cropping strategy

**DOI:** 10.3389/finsc.2023.1233130

**Published:** 2023-08-17

**Authors:** Pin-Chu Lai, Ramandeep Kaur Sandhi, Brian A. Nault

**Affiliations:** Department of Entomology, Cornell University, Cornell AgriTech, Geneva, NY, United States

**Keywords:** *Phytomyza gymnostoma*, leek, scallion, onion, chives, phenology, pest management

## Abstract

Allium leafminer (*Phytomyza gymnostoma* Loew) is an emerging invasive pest of *Allium* crops and has been threatening *Allium* crop production in the eastern United States since its introduction in 2015. *Phytomyza gymnostoma* can cause substantial economic loss in leek crops when late instars tunnel into the lower portion of the plant, which often renders the crop unmarketable. With limited management tools that are cost-effective and practical, especially for leeks produced in organic systems, we examined the attractiveness of other *Allium* crop species that might be considered in a trap cropping strategy. In 2021 and 2022, controlled environment choice tests and field trials were conducted to evaluate host preference of *P. gymnostoma* among *Allium* crop species including chives, scallion, an onion and scallion hybrid, and leek. We also assessed preference of P. gymnostoma for scallions that varied in size/age. Results from field trials indicated that only chives had more oviposition marks, cumulative numbers of eggs, and a higher density of *P. gymnostoma* larvae and pupae than leeks. Larger/older scallions had more oviposition marks and higher *P. gymnostoma* densities than smaller ones in controlled environment choice tests, but this size/age preference was not evident in field trials. Based on our findings, chives could be considered as a potential trap crop for minimizing *P. gymnostoma* damage in leek crops.

## 1 Introduction

Allium leafminer (*Phytomyza gymnostoma* Loew) (Diptera: Agromyzidae) originated from Europe and is now an invasive species in North America (1). Since the first report of *P. gymnostoma* in Pennsylvania, United States, in 2015, *P. gymnostoma* has spread to nearby states on the East coast from New York to Virginia (B. Nault, personal communication). As its common name implies, *P. gymnostoma* is a specialist with a host range limited to the genus *Allium* (family Liliaceae), which includes many important high-value crops grown in the United States, such as onion, garlic, scallion, leek, and chives (1–3). Aesthetic damage occurs when females puncture leaves with its ovipositor, leaving a series of linear white marks (blemishes) that can cause some *Allium* crops to become unmarketable. Eggs are not always laid during this process, rather the oviposition punctures produce plant exudates that are ingested by adults of both sexes. Larval feeding can cause damage to leaves such as mining, distortion and wilting. The most severe damage occurs when late instars feed and pupate at the base of plants. Larval feeding creates entry points for bacterial and/or fungal pathogens to infect the crop, and this can cause the crop to rot. Furthermore, the presence of larvae and pupae within leek plants and garlic cloves at harvest contaminates the crop and can make it unmarketable (1, 2, 4–6).

*Phytomyza gymnostoma* has two generations per year in North America as it does in Europe (1, 3, 4). In the northeastern United States, the spring generation is active from April to May. *Phytomyza gymnostoma* aestivates in the pupal stage during the summer months. Flies from the fall generation emerge in September and remain active through November. Afterward, *P. gymnostoma* overwinters in the pupal stage at the base of plants or in surrounding soil (3). While both generations can cause significant damage to *Allium* crops, the fall generation has a tendency to be more damaging than the spring generation on most farms in New York (B. Nault, personal observation). *Phytomyza gymnostoma* also has been more problematic on organic farms than on conventional farms, which could reflect differences in insecticide use. In addition, some growers stagger plantings of *Allium* crops throughout the year, and this provides access to hosts for both generations of *P. gymnostoma*, which could facilitate population growth on those farms (1). Damage to leek crops on some organic farms in the northeastern United States has been so prevalent and difficult to manage that leeks are no longer grown on these farms. The common name of *P. gymnostoma* in Europe is the leek mining fly, and leek has mostly been reported as the major crop that has suffered from severe damage by *P. gymnostoma* in many European countries including Belgium, Hungary, Italy, and Poland (1, 7–10).

Insecticide use is the most effective tool for managing *P. gymnostoma* infestations. Some of the best conventional products include cyantraniliprole, dinotefuran, and spinetoram, while spinosad is the best product for organic production (11). In addition, Nault et al. (12) demonstrated that *P. gymnostoma* could be managed with only two applications of spinosyn insecticides as long as the applications were made after the first week of initial *P. gymnostoma* detection. While *P. gymnostoma* infestations can be managed effectively with insecticides, growers are seeking alternative non-chemical management strategies that are less expensive and less time-consuming.

Crop rotation has been recommended for home gardens and organic production systems as *P. gymnostoma* overwinters within host plants and adjacent soil and is not known to disperse long distances. However, crop rotation might only be effective if no other *Allium* crops or wild *Allium* hosts are present nearby (1, 13). In some instances, delayed planting of *Allium* crops in the spring after peak adult flight has been successful (10, 13). Nevertheless, delayed planting is not feasible in the fall because infestations occur from September through December. Parasitism from two parasitoid wasps, *Halticoptera circulus* (Walker) and *Chrysocharis oscinidis* Ashmead, has been documented in Pennsylvania, but current parasitism rates have been too low to prevent *P. gymnostoma* from causing economic loss (3).

Trap cropping has not been explored as a management tactic for *P. gymnostoma*. This management tactic involves planting attractive host plants in proximity to the main cash crop in order to lure and concentrate populations of the target pest away from the main crop (14, 15). The foundation of trap cropping considers that insects have a strong preference for one host over another for feeding and reproduction (14). A preferred host could be a particular plant species, cultivar, as well as a particular phenological host stage (14, 16). Hence, one of the first steps for developing a trap cropping approach for *P. gymnostoma* management is to identify an *Allium* crop and a particular phenological stage that are highly attractive to females.

The objective of this study was to identify a highly preferred host for *P. gymnostoma* that could be considered in the future as a potential trap crop, especially for leek production in the fall. Therefore, *Allium* crops commonly grown in the fall such as scallion, an onion and scallion hybrid, and chives were selected as trap crop candidates. In addition, we tested whether plant size/age impacted host preference using small (young) and large (old) scallions. We hypothesized that *P. gymnostoma* would have a distinct preference for one or more of these *Allium* crops and for larger/older plants. Dependent variables used to determine host preference were number of oviposition punctures (for adult feeding and egg deposition) and densities of larvae and pupae. An ideal host for a future trap crop to minimize *P. gymnostoma* damage in leeks would be one that was highly preferred for both egg deposition and larval and pupal establishment.

## 2 Materials and methods

### 2.1 Choice tests in controlled environments

Choice tests were conducted in a glass greenhouse and a walk-in environmental chamber to evaluate *P. gymnostoma* adult host preference among selected *Allium* species and plant sizes/ages at Cornell AgriTech in Geneva, NY in April and May 2022. The greenhouse and the environmental chamber were set at 21°C during the day and 16°C at night with a 14:10 h day/night cycle throughout the study.

#### 2.1.1 *Phytomyza gymnostoma* and plant material preparation

*Phytomyza gymnostoma* used in choice tests were reared from pupae that were extracted from field-collected leek crops the previous fall. Leeks infested by *P. gymnostoma* pupae were collected from the field in November 2021 and stored in a cold room (4.5°C) until April 2022. Pupae then were dipped in a sterilization solution (1% methyl paraben and 2% sodium hypochlorite in deionized water) for 15 seconds and then rinsed with deionized water. Pupae were air-dried on paper towels and then placed in plastic boxes containing moist, autoclaved sand. Pupae were kept in BugDorm cages (60 x 60 x 60 cm; MegaView Science Co., Ltd., Taichung, Taiwan) and incubated in the walk-in environmental chamber. BugDorm cages were supplied with moist cotton balls and greenhouse-grown scallion plants as the food source for newly emerged adult flies. All adults were allowed to mate for at least two days after emergence, and up to five-day old flies were used in the experiments. All plants used in the choice tests were grown from seeds in multi-cell plug flats (Griffin Greenhouse Supplies, Inc., Auburn, NY, USA) in organic potting mix (LM-111 Organic All Purpose Mix, Lambert Peat Moss, Quebec, Canada), and plants were propagated in the glass greenhouse. Plants were fertilized with fish emulsion (2-3-1 N-P-K, Liquid #3, The Fertrell Company, Bainbridge, PA, USA) at a rate of 9.7 ml/L once a week after gemination. Plants were transplanted in plastic pots (10.2 cm in diameter) four weeks after seeding. Plants were at least four weeks old when adult choice tests were initiated.

#### 2.1.2 Experimental design

Four *Allium* crop species were used in the host species preference experiments, which included chives (*Allium schoenoprasum* var. ‘Staro’; Johnny’s seeds, Winslow, ME, USA), leek (*Allium porrum* var. ‘Lancelot’; High Mowing Organic Seeds, Wolcott, VT, USA), scallion (*Allium fistulosum* L., var.’Nabechan F1’; Johnny’s seeds, Winslow, ME, USA), and an onion and scallion hybrid (*Allium fistulosum x A. cepa*, var. ‘Guardsman’; Johnny’s seeds, Winslow, ME, USA). Choice experiments were conducted in BugDorm cages with one plant of each *Allium* species of the same age randomly arranged in each cage. Two mated females were released into each cage. Plant ages varied from seven to nine weeks old across experiments depending on the availability of adult flies and when the experiments were initiated, but plants within each cage were always the same age. Three and four experiments were set up in the greenhouse and the environmental chamber, respectively, from 12-27 April 2022 resulting in a total of seven experiments.

Host size/age choice tests were also conducted in the same controlled environments as the host species preference experiments. Scallions were selected for this study because they are an attractive host and highly susceptible to *P. gymnostoma* (11). Three sizes/ages of scallions were used for the experiments (i.e., four (small), six (medium), eight (large) week-old scallions; the height of the tallest leaf and width of the base of leaves were measured during each experiment and results are shown in **Supplementary Figure 1**). Three experiments were conducted in the greenhouse and another three experiments in the environmental chamber from 18-23 April 2022 resulting in a total of six experiments.

#### 2.1.3 Data collection and statistical analyses

Oviposition marks per plant were recorded every two to four days for three weeks starting one to two days after flies were released into cages. All plants were dissected under a stereo microscope (Zeiss Stemi 2000, 6.5-10X) to obtain numbers of *P. gymnostoma* eggs, larvae, and pupae in each plant at the end of the experiments (~21 days after flies were released). Final numbers of oviposition marks per cm^2^ leaf area and final densities of *P. gymnostoma* (i.e., total number of eggs, larvae, and pupae per plant) were the response variables analyzed for host species and size/age preference. Data from all experiments were pooled within each preference choice test (i.e., host species and size/age preference). Both response variables were analyzed using generalized linear mixed models with the negative binomial distribution and the log link function under PROC GLIMMIX procedure in SAS Studio version 3.81 (Enterprise edition 2022, SAS Institute Inc., Cary, NC, USA). *Allium* species and plant size/age were the fixed factor in two separate analyses, while the controlled environment type (i.e., greenhouse or environmental chamber) was used as a blocking factor that served as a random factor in both analyses. Least squares means (LS-means) were used for post hoc comparisons with Tukey Studentized Range (HSD) Test when the fixed factor was significant at α = 0.05 for mean separation.

### 2.2 Field trials

In 2021, field trials were conducted on commercial organic farms in eastern New York near Hurley (41°55'47.6"N 74°03'58.8"W) and Red Hook (42°01'37.3"N 73°52'15.8"W). In 2022, one trial was conducted on a commercial organic farm in central NY near Fenner (42°58'48.4"N 75°48'48.6"W), while the other trial was on the same farm in Hurley, NY (41°55'50.6"N 74°03'58.4"W). This study was conducted in the fall of both years to take advantage of high *P. gymnostoma* pressure. All field trials were conducted on white plastic mulch with drip irrigation, except in Hurly in 2022 where the trial was conducted on silver reflective mulch with drip irrigation. Organic fertilizer was applied by the cooperating grower prior to transplanting at rates typically used on that farm. No pesticides were applied in our field trials. Infestation by other insect pests and infection by foliar pathogens were minimal both years. Ground cloth (Sunbelt woven ground cover; DeWitt Company, Sikeston, MO, USA) was placed between beds for weed management, and weeds in beds were removed by hand as needed.

#### 2.2.1 Experimental design

Four field trials were conducted in 2021 and 2022 to determine host species preference. The same four *Allium* crop species used in the controlled environment experiments were included in the field trials. The four *Allium* species were arranged in a randomized complete block design with five replications. Each experimental unit (plot) was 7.6 m long and contained three rows of plants in which rows were spaced 0.15 to 0.3 m apart. Plant density was two plants per 0.3 m. Plot beds were spaced 2 m apart and plots were separated within beds by 1-1.5 m wide buffer zones.

Two field trials were conducted in 2022 to determine host size/age preference. Two sizes/ages of scallions were evaluated in which large scallions were eight weeks older and four weeks older than small scallions at Hurley and Fenner, respectively (size measurements in **Supplementary Figure 2**). Large (old) and small (young) plants were arranged in a randomized complete block design with four replications. Each experimental unit (plot) was 3 m long with the same number of rows, plant spacing and bed spacing as described for the host species preference trials. Plots were spaced 0.75 m apart within beds.

#### 2.2.2 Plant material preparation

Seeds for all species were planted in multi-cell plug flats with five seeds per cell in organic potting mix on 21 June for both trials in 2021. In 2022, seeds of all species were planted with three seeds per cell on 16 June and 14 July for trials at Fenner and Hurley, respectively, except that a subset of scallions were planted earlier on 16 May for large size plants in the size preference study. All plants were propagated in glass greenhouses maintained at the same settings as mentioned in the controlled environment experiments. All flats were thinned to one plant per cell two weeks after seeding. Plants were fertilized with fish emulsion once a week after germination as described previously. One to three weeks before transplanting in the field, seedlings were removed from the greenhouse and placed in a shaded area with open air for hardening-off, which prevented plants from going through “transplant shock” by sudden exposure to direct sunlight. Seedlings were transplanted by hand on 20 and 25 August 2021 at Hurley and Red Hook, respectively. In 2022, plants were transplanted on 26 August and 1 September at Fenner and Hurley, respectively.

#### 2.2.3 Data collection

Data collection started within seven days of first detecting either *P. gymnostoma* adults or their oviposition marks on leaves. Twenty-five plants in each plot were randomly selected and visually examined for *P. gymnostoma* oviposition marks. Plants with at least one oviposition mark were considered damaged. Up to ten damaged plants per plot were pulled, and whole plant samples were placed in brown paper grocery bags (1/6 BL; Uline, Pleasant Prairie, WI). Samples were brought back to the laboratory and stored in a cold room (4.5 °C) before further evaluation. All plant samples were processed no more than 14 days after collection from the field. Number of *P. gymnostoma* oviposition marks were counted on each plant. The whole plant was then dissected under a stereo microscope, and the numbers of *P. gymnostoma* eggs, larvae, and pupae were recorded. Plant samples were collected once every two weeks for a total of five sampling events. In 2022, when *P. gymnostoma* damage became extremely high later in the season on chives (i.e., the last two and final sampling dates for Hurley and Fenner, respectively), count data were obtained from only half the leaves from each plant, and data were extrapolated to a per plant basis.

Degree of infestation over time at a plant population level was determined by proportions of damaged plants (i.e., plants with *P. gymnostoma* oviposition marks); the number of plants with *P. gymnostoma* damage was divided by 25 (total number sampled) to obtain the proportion of plants with oviposition marks. Number of oviposition marks per cm^2^ leaf area over time, cumulative number of eggs per plant throughout the season, number of larvae per plant over time, and number of pupae per plant on the last sampling date of the season were used to compare possible feeding and reproduction host preferences among *Allium* species and plant sizes/ages. Number of larvae plus pupae per plant on the sampling date with the highest overall *P. gymnostoma* numbers was an extra response variable used to represent overall *P. gymnostoma* damage on individual plants. In addition, the frequency of eggs deposited by *P. gymnostoma*, determined by percentages of oviposition marks with eggs, was calculated to serve as another response variable for comparing reproduction preference. For simplicity, only data collected on the sampling date with the overall highest number of eggs deposited was used for statistical analysis.

#### 2.2.4 Statistical analyses

The effect of host species and sizes/ages on the numbers of *P. gymnostoma* oviposition marks, eggs, larvae, and pupae were analyzed using generalized linear mixed models with PROC GLIMMIX procedure in SAS Studio version 3.81. Data were analyzed separately by location and year. Repeated measures and the binomial distribution with the logit link function were used to analyze the proportions of plants with oviposition marks; however, when the analysis was not able to converge with the binomial distribution, the Poisson distribution with the log link function was used instead. The beta distribution with the logit link function was used for the frequency of eggs deposited (percentage of oviposition marks with eggs). For count data including oviposition marks, eggs, larvae, pupae, and larvae plus pupae, when normality assumption was met, the Gaussian distribution with the identity link function was used; otherwise, either the negative binomial or the Poisson distribution with the log link function with a lower Akaike information criterion (AIC) value (i.e., a better fit) was used. Across response variables, host plant species/sizes and sampling dates (when applicable) were the fixed factors, while replication was the random factor in the analyses. LS-means were used for post hoc comparisons with Tukey Studentized Range (HSD) Test when the fixed factor was significant at α = 0.05 for mean separation. In the analysis for the effect of host plant species on number of pupae per plant on the last sampling date at Fenner in 2022, T-grouping was used for mean separation as Tukey Studentized Range (HSD) Test was too conservative to separate LS-means.

## 3 Results

### 3.1 Host plant species preference in controlled environment choice tests

**Figure 1:**
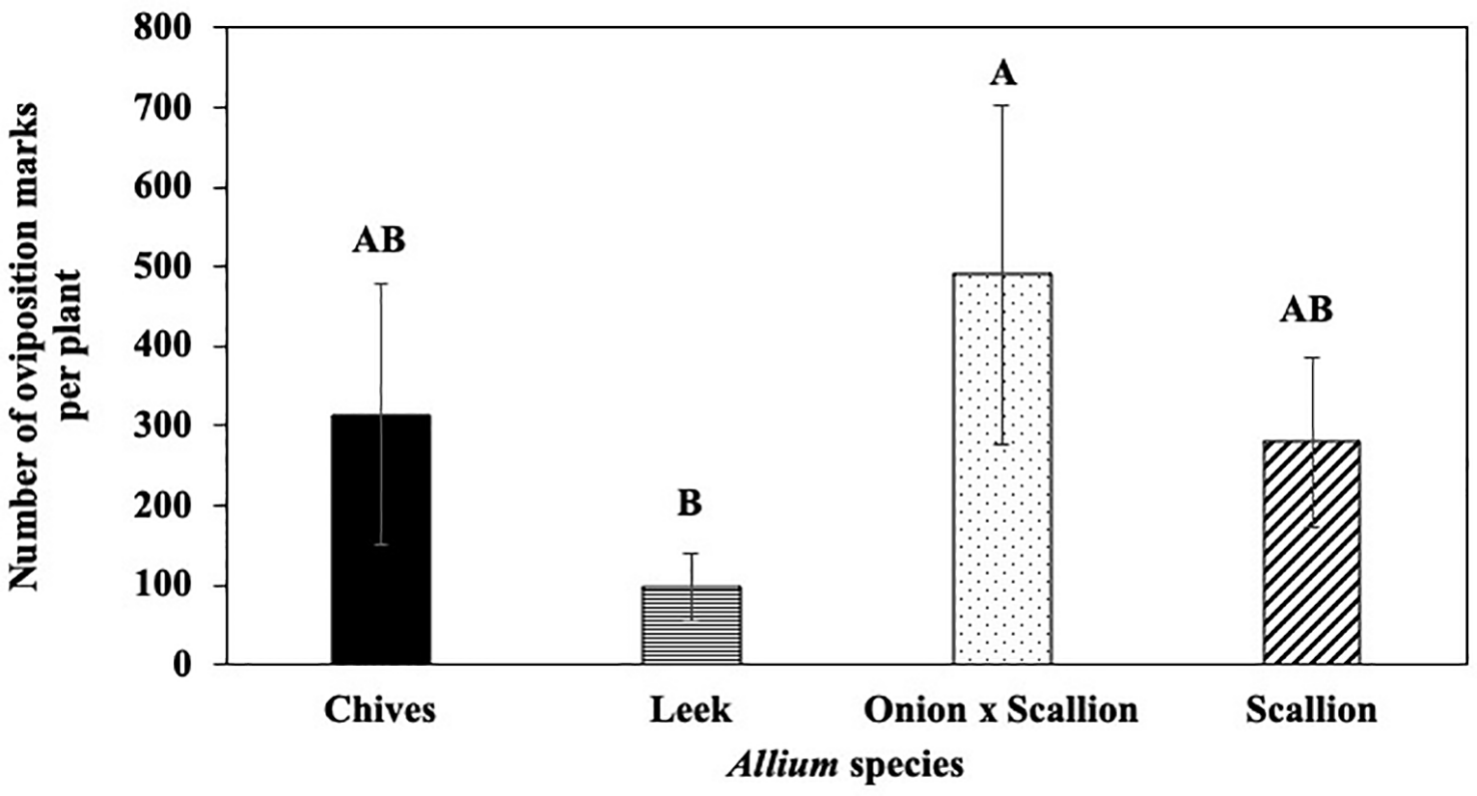
Mean (± SE) number of *Phytomyza gymnostoma* oviposition marks per plant at 21 days after flies were released in controlled environment choice tests. Different letters indicate significant differences in least squares means of number of oviposition marks among *Allium* species (Tukey-HSD; P < 0.05; n = 7).

In host species choice experiments, numbers of *P. gymnostoma* oviposition marks per plant varied among *Allium* species (F_3, 18_ = 3.61, P = 0.034). The onion and scallion hybrid had more oviposition marks than leeks, while numbers of marks in chives and scallions were not statistically different from leeks, but all were numerically higher than leeks (**Figure 1**). However, total number of *P. gymnostoma* (eggs, larvae, and pupae combined) per plant at 21 days after flies were released were not different among *Allium* species (P = 0.244; **Supplementary Figure 3**).

### 3.2 Host plant size/age preference in controlled environment choice tests

**Figure 2:**
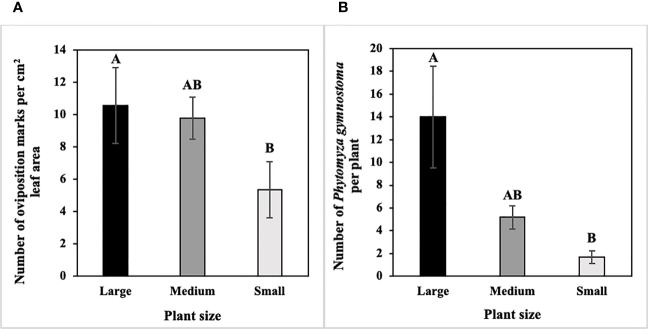
Mean (± SE) number of *Phytomyza gymnostoma* **(A)** oviposition marks per cm^2^ leaf area and **(B)** eggs, larvae, and pupae combined per plant at 21 days after flies were released in controlled environment choice tests. Different letters within each graph indicate significant differences in least squares means of number of oviposition marks or total number of *P. gymnostoma* among plant sizes (Tukey-HSD; P < 0.05; n = 6).

In host size/age choice tests, numbers of *P. gymnostoma* oviposition marks per cm^2^ leaf area differed among host sizes/ages (F_2, 10_ = 4.93, P = 0.032). Large scallions had more oviposition marks per cm^2^ leaf area than small scallions, and numbers of marks per cm^2^ leaf area in medium scallions were not different from large or small scallions (**Figure 2A**). In addition, densities of *P. gymnostoma* also differed among host sizes/ages at 21 days after flies were released (F_2, 10_ = 11.39, P = 0.003). Large scallions had more *P. gymnostoma* (eggs, larvae, and pupae combined) per plant than small scallions, while *P. gymnostoma* densities in medium scallions were not different from large or small scallions (**Figure 2B**).

### 3.3 Host plant species preference in field trials

**Figure 3:**
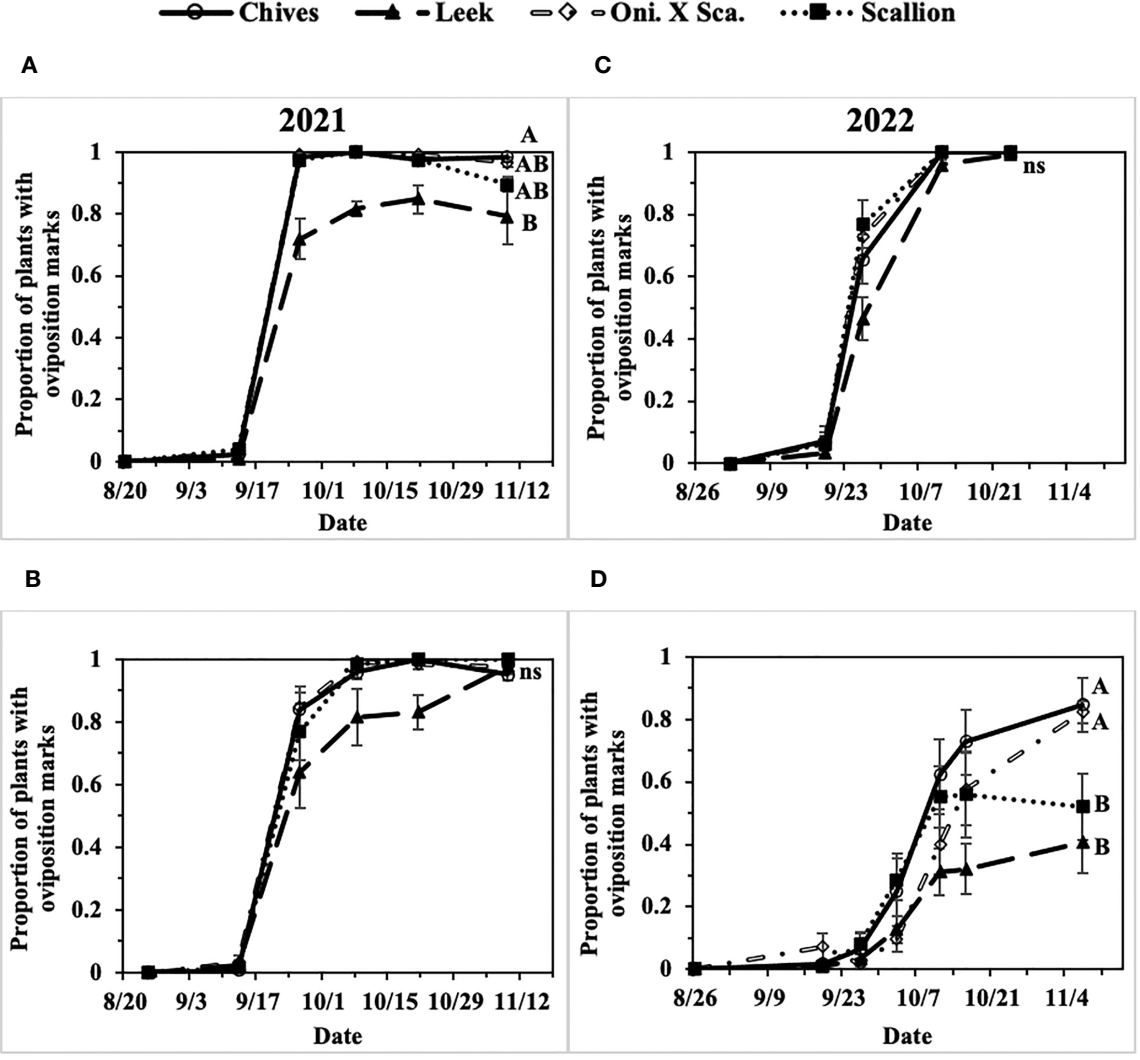
Mean (± SE) proportion of plants with *Phytomyza gymnostoma* oviposition marks over time in **(A)** Hurley, NY, 2021, **(B)** Red Hook, NY, 2021, **(C)** Hurley, NY, 2022, and **(D)** Fenner, NY, 2022. Different letters within each graph indicate significant differences in least squares means of proportion of plants with oviposition marks among *Allium* species on the final sampling date (Tukey-HSD; P < 0.05; n = 5); an ‘ns’ indicate no significant difference (P > 0.05) among *Allium* species on the final sampling date. “Oni. X Sca.” in the legend denotes the onion and scallion hybrid.

In the two-year field study, extremely high populations of *P. gymnostoma* were observed in field sites in eastern NY in 2021 and 2022, whereas moderate population pressure was observed in central NY in 2022. In 2021 at Hurley and Red Hook, 91% and 98% of plants were considered damaged (i.e., presence of oviposition marks) by the end of the season across all *Allium* species (**Figures 3A, B**). In 2022 at Hurley, the overall plant damage was nearly 100% averaged across all *Allium* species, whereas at Fenner the overall plant damage was 65% averaged across all *Allium* species (**Figures 3C, D**).

#### 3.3.1 Progression of *P. gymnostoma* infestations in *Allium* crops

The first activity of fall-generation *P. gymnostoma* in eastern New York (Hurley, NY) was reported on 7 and 14 September of 2021 and 2022, respectively. Progression of *P. gymnostoma* infestations at a plant population level, represented as proportions of plants with oviposition marks, followed sigmoid curves with less than 10% of plants with oviposition marks within the first week after initial detection of activity. In general, infestations of *P. gymnostoma* progressed more slowly in leeks than in chives, scallions, and the onion and scallion hybrid (**Figure 3**).

In 2021 at both locations, infestation levels increased to over 50% within three weeks after initial detection in all *Allium* species (**Figures 3A, B**). At the end of the season at Hurley, there were significantly lower proportions of leeks with oviposition marks than chives (F_3, 16_ = 4.62, P = 0.016; **Figure 3A**). While at Red Hook, almost all plants had *P. gymnostoma* oviposition marks regardless of *Allium* species (P = 0.787; **Figure 3B**).

In 2022 at Hurley, infestation levels increased over 50% within two weeks in chives, scallions, and the onion and scallion hybrid, but not in leeks. At the end of the season, almost all plants had *P. gymnostoma* oviposition marks regardless of *Allium* species (P = 0.999; **Figure 3C**). In 2022 at Fenner, *P. gymnostoma* infestation levels progressed more slowly and did not exceed 50% infestation until four to five weeks after initial detection of activity in chives, scallions, and the onion and scallion hybrid, while the level of infestation in leeks never exceeded 50%. At the end of the season, there were significantly lower proportions of leeks and scallions with oviposition marks than chives and the onion and scallion hybrid (F_3, 10.13_ = 11.99, P = 0.001; **Figure 3D**).

#### 3.3.2 Number of *P. gymnostoma* oviposition marks and eggs (*Allium* species)

**Figure 4:**
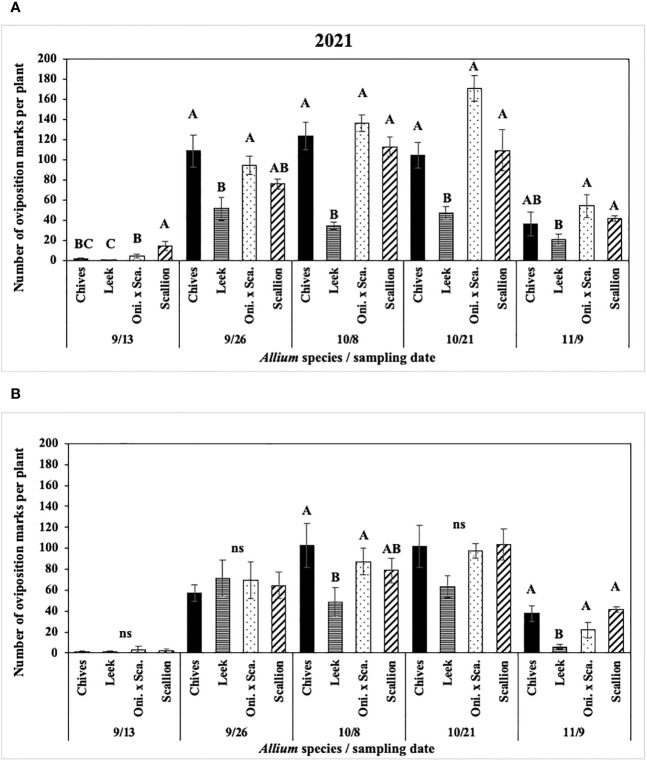
Mean (± SE) number of *Phytomyza gymnostoma* oviposition marks per plant on each of five sampling dates in **(A)** Hurley, NY and **(B)** Red Hook, NY in 2021. Different letters within each sampling date of each graph indicate significant differences in least squares means of number of oviposition marks among *Allium* species on each of the sampling date (Tukey-HSD; P < 0.05; n = 5), while ‘ns’ indicates no significant differences (P > 0.05) among *Allium* species. “Oni. x Sca.” on the x-axes denotes the onion and scallion hybrid.

In 2021 at both locations, numbers of *P. gymnostoma* oviposition marks per plant differed among *Allium* species and sampling dates with a significant interaction effect (Hurley: F_12, 76_ = 5.14, P < 0.001; Red Hook: F_12, 76_ = 3.62, P < 0.001; **Figure 4**). At Hurley, scallions had the highest number of oviposition marks on the first sampling date. Numbers of oviposition marks on chives and scallions were higher than those on leeks on three of the four sampling dates after the first sampling date, while number of marks on the onion and scallion hybrid was consistently higher than those on leeks after the first sampling (**Figure 4A**). At Red Hook, numbers of oviposition marks were not different among *Allium* species on three of five sampling dates; however, the number of oviposition marks on chives and the onion and scallion hybrid was significantly greater than on leek on 8 October (the middle sampling date), and there were more oviposition marks on chives, scallions, and the hybrid than on leek on the last sampling date. (**Figure 4B**).

**Figure 5:**
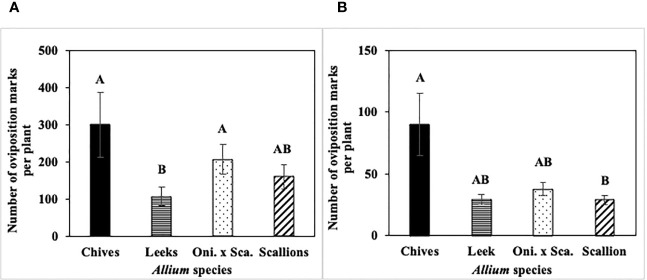
Mean (± SE) number of *Phytomyza gymnostoma* oviposition marks per plant across the season in **(A)** Hurley, NY and **(B)** Fenner, NY in 2022. Different letters within each graph indicate significant differences (P < 0.05) in least squares means of number of oviposition marks among *Allium* species (Tukey-HSD; P < 0.05; n = 5). “Oni. x Sca.” on the x-axes denotes the onion and scallion hybrid. Note that y-axis scales differ between locations.

In 2022 at both locations, the interaction effect between *Allium* species and sampling dates on numbers of *P. gymnostoma* oviposition marks per plant was not significant (Hurley: P = 0.10; Fenner: P = 0.11). Mean numbers of oviposition marks across all sampling dates differed among *Allium* species. At Hurley, leeks had significantly lower numbers of oviposition marks than chives and the onion and scallion hybrid (F_3, 60_ = 7.19, P < 0.001; **Figure 5A**). At Fenner, scallions had significantly lower numbers of oviposition marks than chives, while number of marks on leeks and the onion and scallion hybrid was not different from those on scallions or chives (F_3, 60_ = 3.25, P = 0.028; **Figure 5B**).

**Figure 6:**
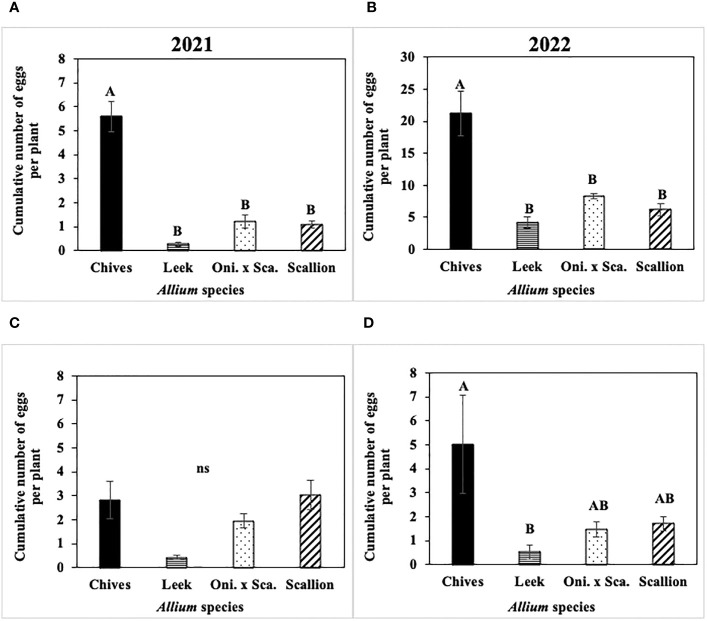
Mean (± SE) cumulative number of *Phytomyza gymnostoma* eggs per plant across the season in **(A)** Hurley 2021, NY, **(B)** Hurley 2022, NY, **(C)** Red Hook 2021, NY, and **(D)** Fenner 2022, NY. Different letters within each graph indicate significant differences in least squares means of cumulative number of eggs per plant among *Allium* species (Tukey-HSD; P < 0.05; n = 5), while ‘ns’ indicates no significant differences (P > 0.05) among *Allium* species. “Oni. x Sca.” on the x-axes denotes the onion and scallion hybrid. Note that the y-axis scale in **(B)** differs from those in **(A, C**, **D)**.

Cumulative number of *P. gymnostoma* eggs per plant across the season differed among *Allium* species in three of four trials (**Figure 6**). In both 2021 and 2022 at Hurley, chives had significantly higher numbers of eggs than leeks, scallions, and the onion and scallion hybrid (2021: F_3, 12_ = 9.67, P = 0.002, **Figure 6A**; 2022: F_3, 16_ = 20.31; P < 0.001, **Figure 6B**). In 2021 at Red Hook, no difference was found in egg numbers among species (P = 0.08; **Figure 6C**). In 2022 at Fenner, chives had significantly higher numbers of eggs than leeks (F_3, 16_ = 4.83; P = 0.014; **Figure 6D**).

**Figure 7:**
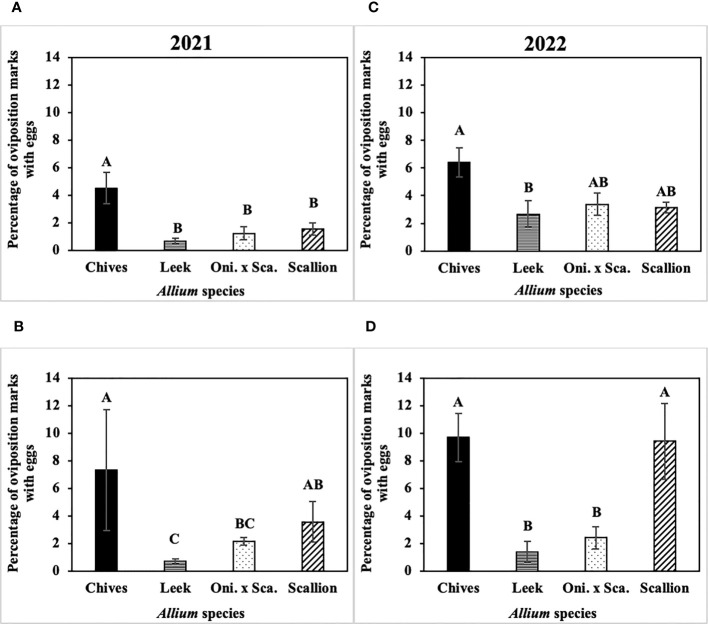
Mean (± SE) percentage of *Phytomyza gymnostoma* oviposition marks with eggs on the sampling date with the highest overall number of eggs during the season in **(A)** Hurley, NY on 26 September 2021, **(B)** Red Hook, NY on 26 September 2021, **(C)** Hurley, NY on 26 September 2022, and **(D)** Fenner, NY on 3 October 2022. Different letters within each graph indicate significant differences in least squares means of percentage of oviposition marks with eggs among *Allium* species (Tukey-HSD; P < 0.05; n = 5). “Oni. x Sca.” on the x-axes denotes the onion and scallion hybrid.

The frequency of *P. gymnostoma* eggs deposited while making the same number of oviposition marks differed among *Allium* species (**Figure 7**). In 2021 at Hurley, the percentage of oviposition with eggs was higher in chives than in leeks, scallions, and the onion and scallion hybrid (F_3, 11_ = 8.74; P = 0.003; **Figure 7A**); while in 2021 at Red Hook, the percentage of oviposition with eggs was higher in chives than in leeks and the hybrid (F_3, 12_ = 7.27; P = 0.005; **Figure 7B**). In 2022 at Hurley, the percentage of oviposition with eggs in chives was higher than in leeks (F_3, 12_ = 4.21; P = 0.023; **Figure 7C**); while in 2022 at Fenner, the percentage of oviposition with eggs in chives and scallions were higher than in leeks and the onion and scallion hybrid (F_3, 9_ = 5.26; P = 0.023; **Figure 7D**). Across *Allium* species and trials, *P. gymnostoma* averaged laying eggs 4% of the time when they made oviposition marks.

Overall, chives had more *P. gymnostoma* oviposition marks and eggs than the other *Allium* species, while leeks had the fewest.

#### 3.3.3 Number of *P. gymnostoma* larvae and pupae (*Allium* species)

**Figure 8:**
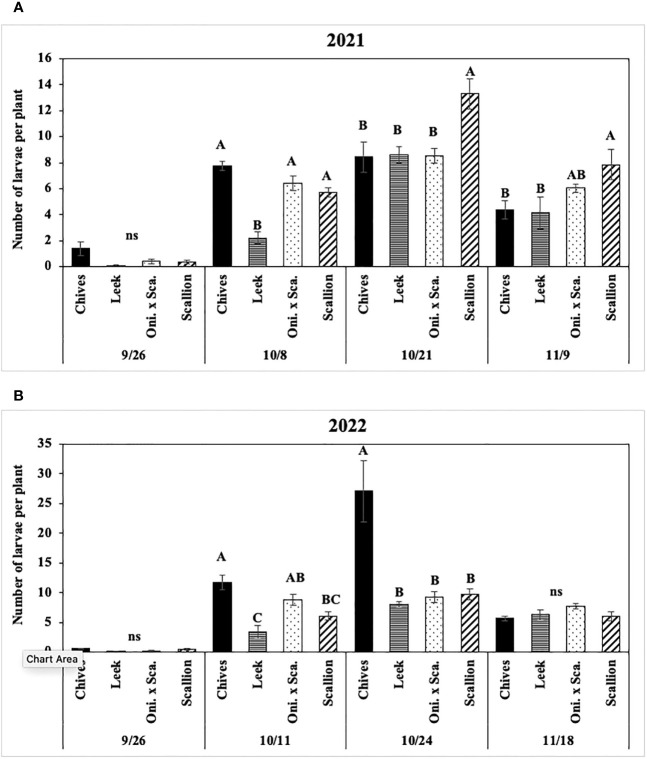
Mean (± SE) number of *Phytomyza gymnostoma* larvae per plant on each of four sampling dates in Hurley, NY in **(A)** 2021 and **(B)** 2022. Different letters within each sampling date of each graph indicate significant differences in least squares means of number of larvae per plant among *Allium* species (Tukey-HSD; P < 0.05; n = 5), while ‘ns’ indicates no significant differences (P > 0.05) among *Allium* species. “Oni. x Sca.” on the x-axes denotes the onion and scallion hybrid. Note that the y-axis scales differ between two years.

**Figure 9:**
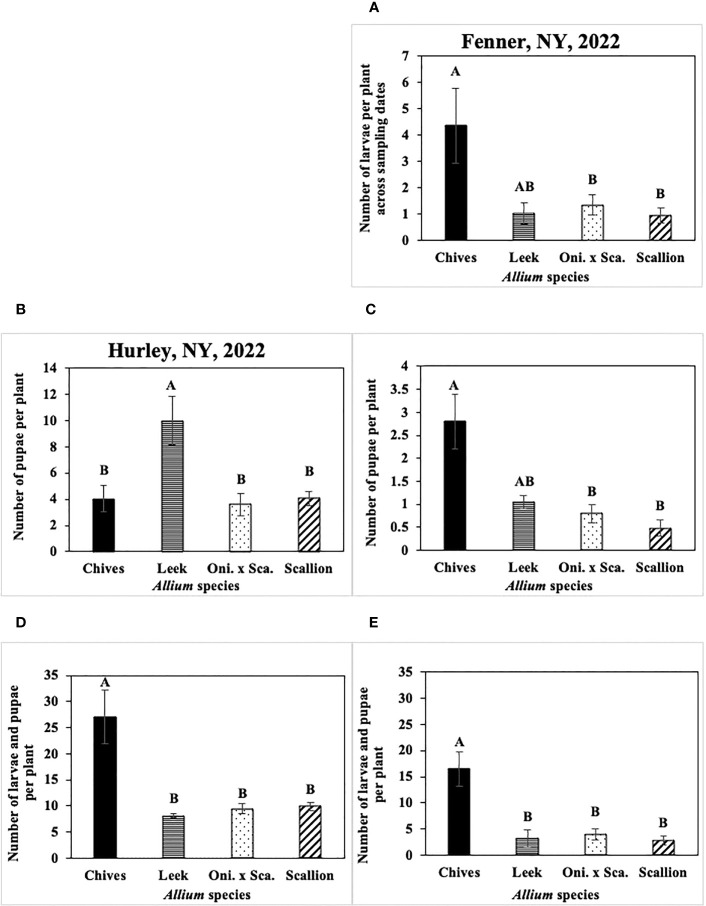
**(A)** Mean (± SE) number of *Phytomyza gymnostoma* larvae per plant across sampling dates in Fenner, NY in 2022. Mean (± SE) number of *P. gymnostoma* pupae per plant on the last sampling date in **(B)** Hurley, NY on 09 November 2022 and **(C)** Fenner, NY on 18 November 2022. Mean (± SE) number of *P. gymnostoma* larvae and pupae per plant on the sampling date with the highest overall number in **(D)** Hurley, NY on 24 October 2022 and **(E)** Fenner, NY on 7 November 2022. Different letters within each graph indicate significant differences in least squares means of number of *P. gymnostoma* per plant among *Allium* species (Tukey-HSD; P < 0.05; n = 5). “Oni. x Sca.” on the x-axes denotes the onion and scallion hybrid. Note that the y-axis scales differ among parameters and locations.

Numbers of *P. gymnostoma* larvae per plant were affected by *Allium* species in three of four trials (**Figures 8**, **9A**). In both years at Hurley, numbers of larvae per plant differed among *Allium* species and sampling dates with a significant interaction effect (2021: F_9, 60_ = 2.44, P = 0.019; 2022: F_9, 60_ = 3.86, P < 0.001; **Figure 8**). In 2021 at Hurley, while the number of larvae did not differ among species on 26 September, the number of larvae in leek was significantly lower than the numbers in the other three species on 8 October; numbers of larvae in leeks were significantly lower than those in scallions, but were similar to those in chives and the onion and scallion hybrid on the latter two sampling dates (**Figure 8A**). In 2022 at Hurley, numbers of larvae were not different among species on 26 September and 18 November; however, the number of larvae in leeks was lower than those in chives and the onion and scallion hybrid, but not different from scallions on 11 October; numbers of of larvae were lower in leek, scallions and the onion and scallion hybrid than in chives on 24 October. (**Figure 8B**).

In 2021 at Red Hook, the effect of *Allium* species on numbers of larvae was not significant (P = 0.767). In 2022 at Fenner, numbers of larvae per plant differed among *Allium* species without a significant interaction effect between species and sampling date. Chives had significantly higher numbers of larvae than scallions and the onion and scallion hybrid across all sampling dates, while numbers of larvae in leeks were similar to those in the other species (F_3, 76_ = 48.36, P < 0.001; **Figure 9A**).

Numbers of *P. gymnostoma* pupae per plant on the last sampling date differed among *Allium* species in two of four trials. In 2021 at Hurley and Red Hook, the effect of *Allium* species on numbers of pupae was not significant (Hurley: P = 0.316; Red Hook: P = 0.199). In 2022 at Hurley, leeks had significantly higher numbers of pupae than chives, scallions, and the onion and scallion hybrid (F_3, 12_ = 7.96, P = 0.004; **Figure 9B**). In 2022 at Fenner, chives had significantly higher numbers of pupae than scallions and the onion and scallion hybrid, while numbers of pupae in leeks were similar to those in the other species (F_3, 12_ = 3.58, P = 0.047; **Figure 9C**).

Numbers of *P. gymnostoma* larvae plus pupae per plant on the sampling date with the highest overall numbers also differed among *Allium* species in two of four trials. In 2021 at Hurley and Red Hook, the effect of *Allium* species on numbers of larvae and pupae was not significant (Hurley: P = 0.083; Red Hook: P = 0.269). In 2022 at Hurley and Fenner, chives had significantly higher numbers of larvae plus pupae than those in leeks, scallions, and the onion and scallion hybrid (Hurley: F_3, 12_ = 26.96, P < 0.001, **Figure 9D**; Fenner: F_3, 12_ = 26.56, P < 0.001, **Figure 9E**).

Overall, numbers of *P. gymnostoma* in plants throughout the season largely varied among *Allium* species and trials. However, chives had higher numbers of larvae plus pupae than the other species late in the season, suggesting that chives is a preferred reproductive host among the species evaluated. Alternatively, there was a higher probability of *P. gymnostoma* survival to pupation in chives compared with the other species.

### 3.4 Host plant size/age preference in field trials

#### 3.4.1 Progression of *P. gymnostoma* infestations (size/age)

**Figure 10:**
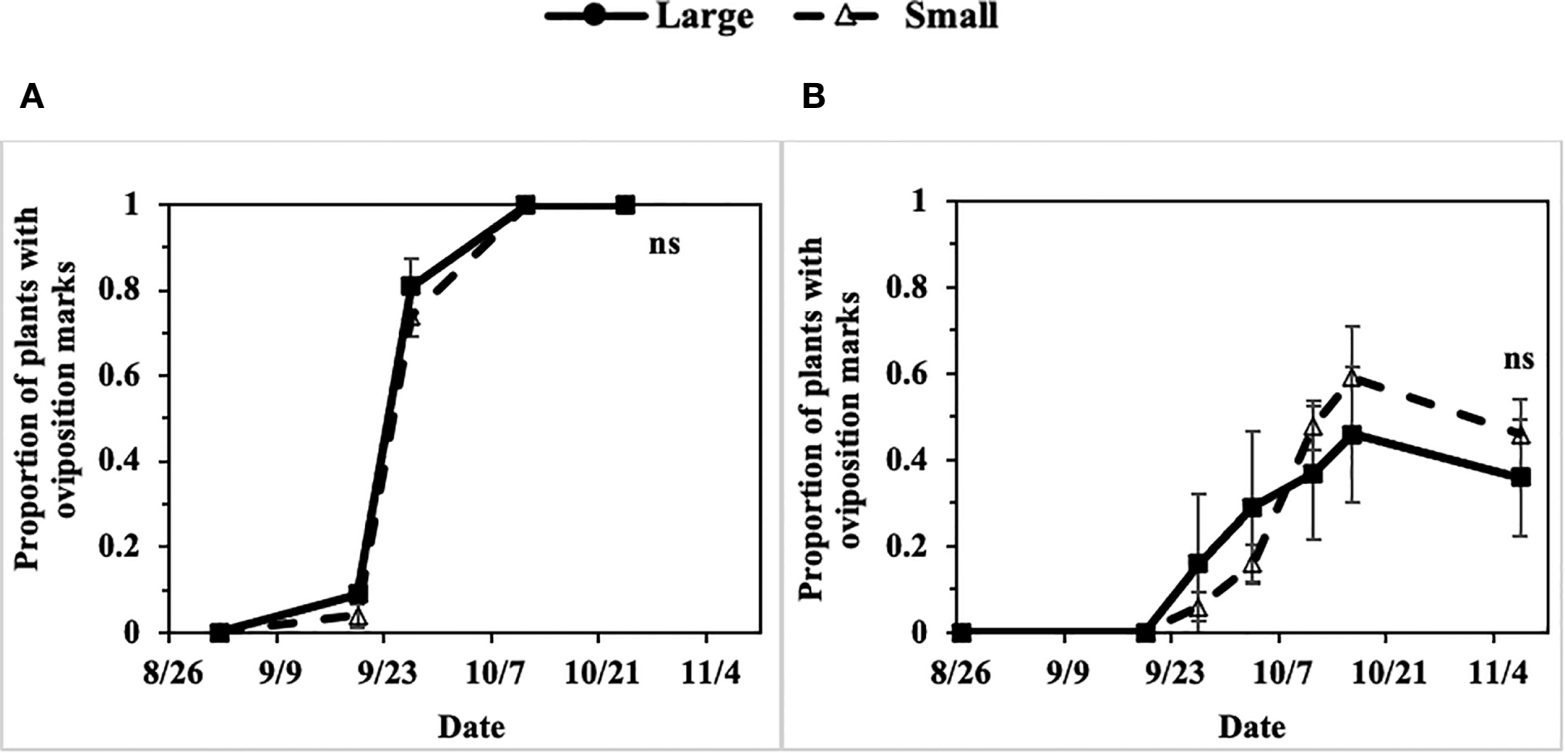
Mean (± SE) proportion of large and small scallions with *Phytomyza gymnostoma* oviposition marks over time in **(A)** Hurley, NY and **(B)** Fenner, NY in 2022. An ‘ns’ indicates no significant differences (Tukey-HSD; P > 0.05; n = 4) in least squares means of proportion of plants with oviposition marks between large and small scallions on the last sampling date.

Progression of the *P. gymnostoma* infestations at a plant population level, represented as proportions of plants with oviposition marks, followed sigmoid curves with no apparent difference in progression trend between large (older) and small (younger) scallions. In 2022 at Hurley and Fenner, the final proportions of plants with oviposition marks were not different between large and small scallions (Hurley: P = 0.991, Fenner: P = 0.494; **Figure 10**).

#### 3.4.2 Number of *P. gymnostoma* oviposition marks and eggs (size/age)

**Figure 11:**
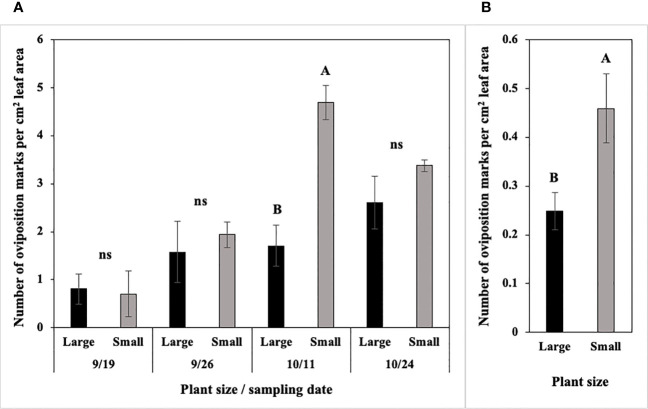
Mean (± SE) number of *Phytomyza gymnostoma* oviposition marks per cm^2^ leaf area **(A)** on four sampling dates at Hurley, NY and **(B)** across sampling dates at Fenner, NY. Different letters within each graph indicate significant differences in least squares means of number of oviposition marks between plant size (Tukey-HSD; P < 0.05; n = 4), while ‘ns’ indicates no significant differences. Note that the y-axis scales differ between two locations.

In 2022 at Hurley, the number of *P. gymnostoma* oviposition marks per cm^2^ leaf area differed by plant size and sampling dates with a significant interaction effect (F_3, 21_ = 6.02, P = 0.004; **Figure 11A**). Small scallions had significantly more oviposition marks per cm^2^ leaf area than large scallions on 11 October, while no differences existed between the two treatments on the other three sampling dates (**Figure 11A**). In 2022 at Fenner, where the overall pressure was lower than at Hurley, the number of *P. gymnostoma* oviposition marks per cm^2^ leaf area also differed by plant size, but without a significant interaction effect with sampling date (F_1, 21_ = 7.31, P = 0.013). The number of oviposition marks per cm^2^ leaf area in smaller scallions was significantly higher than those in larger scallions across sampling dates (**Figure 11B**).

Cumulative number of *P. gymnostoma* eggs per plant was not statistically different between large and small scallions in either trial (Hurley: P = 0.417, Fenner: P = 0.904). However, small scallions tended to have a numerically higher cumulative numbers of eggs than large scallions in both trials (**Supplementary Figures 4A, B**).

Similarly, the frequency of *P. gymnostoma* eggs deposited did not differ by plant size/age in either trial (Hurley: P = 0.088, Fenner: P = 0.247). Small scallions tended to have numerically higher percentages of oviposition marks with eggs than large scallions in both trials (**Supplementary Figures 4C, D**).

Overall, smaller scallions had significantly higher oviposition marks per cm^2^ leaf area and numerically higher number of eggs per plant and frequency of egg deposition, indicating a preference for smaller plants over larger ones.

#### 3.4.3 Number of *P. gymnostoma* larvae and pupae (size/age)

In 2022 at Hurley and Fenner, numbers of *P. gymnostoma* larvae across all sampling dates, numbers of pupae on the last sampling date, and numbers of larvae plus pupae on the sampling date with the highest total *P. gymnostoma* number did not differ by plant size/age [larvae: P = 0.124 (Hurley), P = 0.988 (Fenner); pupae: P = 0.267 (Hurley), P = 0.980 (Fenner); larvae plus pupae: P = 0.058 (Hurley), P = 0.935 (Fenner)]. However, a consistent numerical trend was observed that large scallions had numerically more larvae, pupae, and larvae plus pupae than small scallions (larvae: **Supplementary Figures 4E, F**; pupae: **Supplementary Figures 4G, H**; larvae plus pupae: **Supplementary Figures 4I, J**).

Overall, *P. gymnostoma* larval and pupal densities were statistically similar between large (old) and small (young) plants. However, there was a consistent numerical trend for larger (older) scallions to support more larvae and pupae than smaller (younger) ones. These results likely reflect the ability of more larvae to survive and complete development to the pupal stage on a larger plant compared with smaller ones.

## 4 Discussion

The most important component of a trap crop strategy for pest management is to identify a highly attractive host plant that the target pest distinctly prefers to feed and reproduce on compared with the main crop so that the main crop will avoid or suffer less damage. Our study suggested that *P. gymnostoma* prefers to feed and reproduce on chives more than leeks because chives had more oviposition marks, eggs, larvae and pupae than leeks. Therefore, chives could be considered as a potential trap crop for *P. gymnostoma* management in leek production. Chives were considered highly suitable host plants for *P. gymnostoma* in Austria because they remain in a vegetative stage during both spring and fall generations (17). In our study, we also observed that chives remained in a vegetative stage longer than the other *Allium* crop species as new leaves were continually being produced. *Phytomyza gymnostoma* made oviposition marks and laid eggs in chives more than they did in leeks and other *Allium* species examined, especially towards the end of season. New leaves are often softer and thinner than old leaves, which might have played a role in why *P. gymnostoma* made so many oviposition punctures.

Host feeding and oviposition preference of *P. gymnostoma* has not been investigated elsewhere, and preference can be affected by single or multiple physical and/or chemical factors. Host feeding selection of pea leafminer (*Liriomyza huidobrensis*), another Agromyzid, was positively correlated with moisture content of leaves and negatively correlated with the thickness of the epidermis wall; thickness of the epidermis wall can serve as a physical barrier to female oviposition (18). Chemical content of host leaves could also affect host preference of leaf mining flies. For example, the American serpentine leafminer (*L. trifolii*) preferred to feed and oviposit on tomato plants with a higher nitrogen content, and performance variables of the larvae such as development rate, survivorship, and pupal size were increased with increasing plant nitrogen levels (19). The physical and chemical characteristics of *Allium* species in this study were not investigated, except for visual observation of leaf texture and thickness. While the underlying mechanism of host species preference of *P. gymnostoma* is not known, this is an important topic for future studies to consider. Understanding such a mechanism could be useful for breeding programs seeking to develop resistant cultivars.

Attractiveness of host plants could come from other physiological characteristics such as plant sizes or ages. In our controlled environment experiments, *P. gymnostoma* preferred to make oviposition marks on larger/older scallions than on smaller/younger scallions. Larger/older plants have more leaf area and tissue than smaller plants, which likely makes larger/older plants easier to be visually and/or chemically detected by *P. gymnostoma* if visual and/or chemical cues are important for host-finding. However, *P. gymnostoma* preferred to make oviposition marks on smaller plants than on larger ones in the field. Leaves of scallions in our controlled environment studies were in a relatively young vegetative stage, whereas scallions in the field trials were much older and leaves likely became tougher and thicker over time. Ovipositional preference for smaller plants could be influenced by the softer leaves of smaller plants rather than plant size. In addition, plants grew faster in the field under suitable conditions than in controlled environments. As a consequence, smaller plants tended to catch up phenologically with the larger/older plants more quickly in the field. Large and small plants in our field trials might not have been different enough to trigger a host preference, especially in 2022 at Fenner where large and small scallions were seeded only four weeks apart. Overall, *P. gymnostoma*’s preference for larger/older plants as hosts was only supported by our laboratory studies, but not by our field studies. Therefore, host size/age alone should not be considered as an important factor when designing a trap crop strategy for this pest.

Trap cropping has been evaluated for management of *Liriomyza* spp. in shallot and onion crops. Trap cropping significantly reduced leafminer populations by 40%, and population reduction was up to 48% when trap cropping was integrated with the use of arbuscular mycorrhizae (20). Moreover, trap cropping also attracted and preserved parasitoid wasps as natural enemies in the cropping system in addition to reducing leafminer infestation (21). For developing a trap cropping strategy for *P. gymnostoma* management in leek production in the northeastern United States, our study showed that chives are the best candidate trap crop among the *Allium* species we evaluated. Future studies should focus on determination of a spatial pattern and configuration of planting a trap crop as well as planting distance from the main crop. We observed a relatively pronounced edge effect of *P. gymnostoma* infestation and damage where higher numbers of flies and levels of damage occurred on plants nearest to the previous crop that *P. gymnostoma* had infested. This pattern of crop colonization would lend itself nicely to placing a trap crop between the current season’s crop and the preceding one. Chives (trap crop) could be planted between the preceding *Allium* crop and the current season’s leek crop (main crop) in order to intercept flies in the chives before reaching the leeks (15). Future studies should also look into the impact of using chives as a trap crop in leek production on natural enemy population such as parasitoid wasps.

In addition to host preference of *P. gymnostoma* among common *Allium* crops species and between plant sizes, our study also provided knowledge of *P. gymnostoma* infestation progression in *Allium* crops grown in northeastern United States, which can be useful information for developing and optimizing *P. gymnostoma* management in the field. For example, physical control methods such as exclusion netting and vacuuming should take place soon after initial detection of *P. gymnostoma* because eggs were found as early as five days after initial detection, and infestation reached 100% in the field in two to three weeks under heavy fly pressure in our study.

In conclusion, identifying chives as a potential trap crop that can be explored for reducing *P. gymnostoma* damage in leeks was a promising initial step towards development of an alternative management approach to insecticide use. Our study demonstrated that *P. gymnostoma* preferred chives over leeks for feeding and reproduction. However, the level of preference is not likely strong enough for trap cropping to be effective on its own. Besides trap cropping, cultural practices such as reflective mulch, exclusion netting, and vacuuming are management tactics that have potential for *P. gymnostoma* management, but need to be evaluated. Integrating one or more cultural practices with trap cropping could potentially create synergism to provide an economically acceptable level of *P. gymnostoma* control in leek crops.

## Data availability statement

The original contributions presented in the study are included in the article/**Supplementary Material**. Further inquiries can be directed to the corresponding authors.

## Author contributions

BN, PL, and RS contributed to conception and design of the study. BN acquired the funding for this study. PL and RS executed experiments, collected data, and organized the database. PL performed the statistical analysis. PL wrote the first draft of the manuscript. All authors contributed to the article and approved the submitted version.
