# Supplementary material for: Allium leafminer (Diptera: Agromyzidae) host preference: implications for developing a trap cropping strategy
Source: Front Insect Sci. 2023 Aug 17;3:1233130. doi: 10.3389/finsc.2023.1233130 (PMC10926477; doi:10.3389/finsc.2023.1233130)
Supplement: Supplementary file 1 [file DataSheet_1.pdf]

Supplementary Material

Allium leafminer (Diptera: Agromyzidae) host preference: implications for developing a trap cropping strategy

Pin-Chu Lai*, Ramandeep Kaur Sandhi, Brian A. Nault

*** Correspondence:** Corresponding Author: pl484@cornell.edu

1 Supplementary Figures

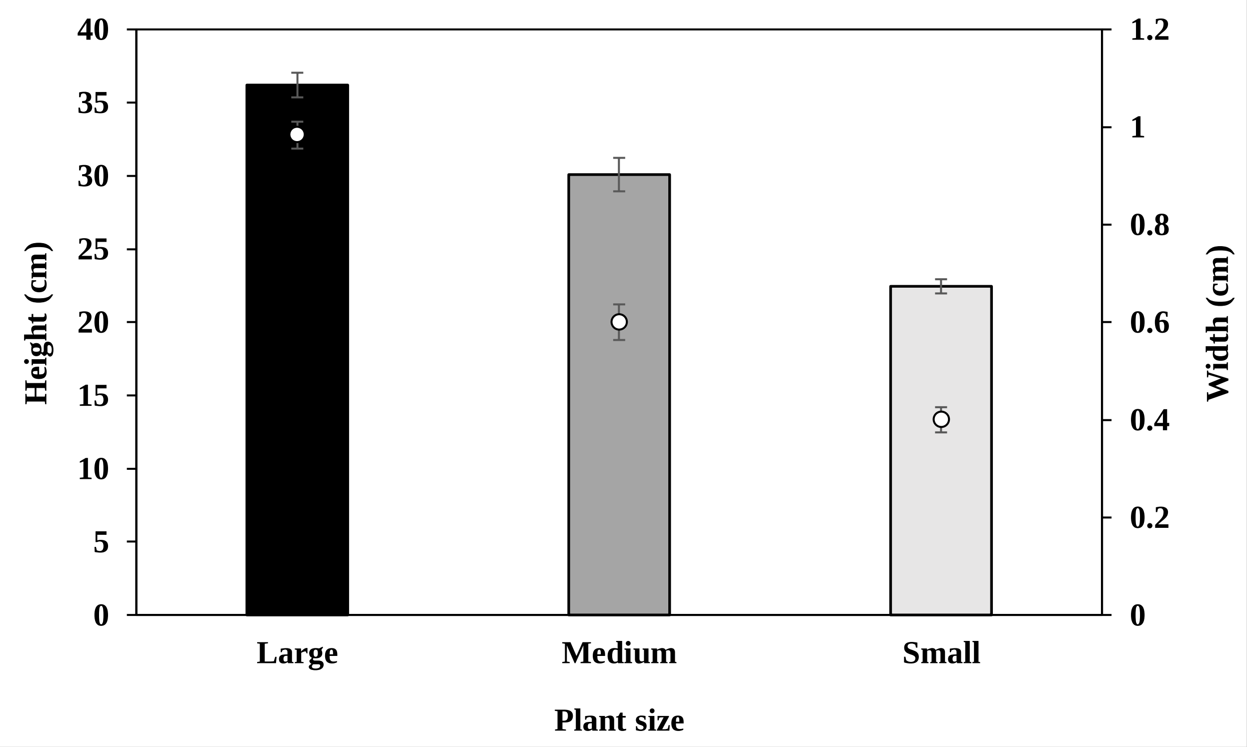

Supplementary Figure 1. Mean (± SE) height in bars and mean (± SE) width of the base of the leaves in dots of large, medium, and small scallions at the end of the size choice tests in controll environments.

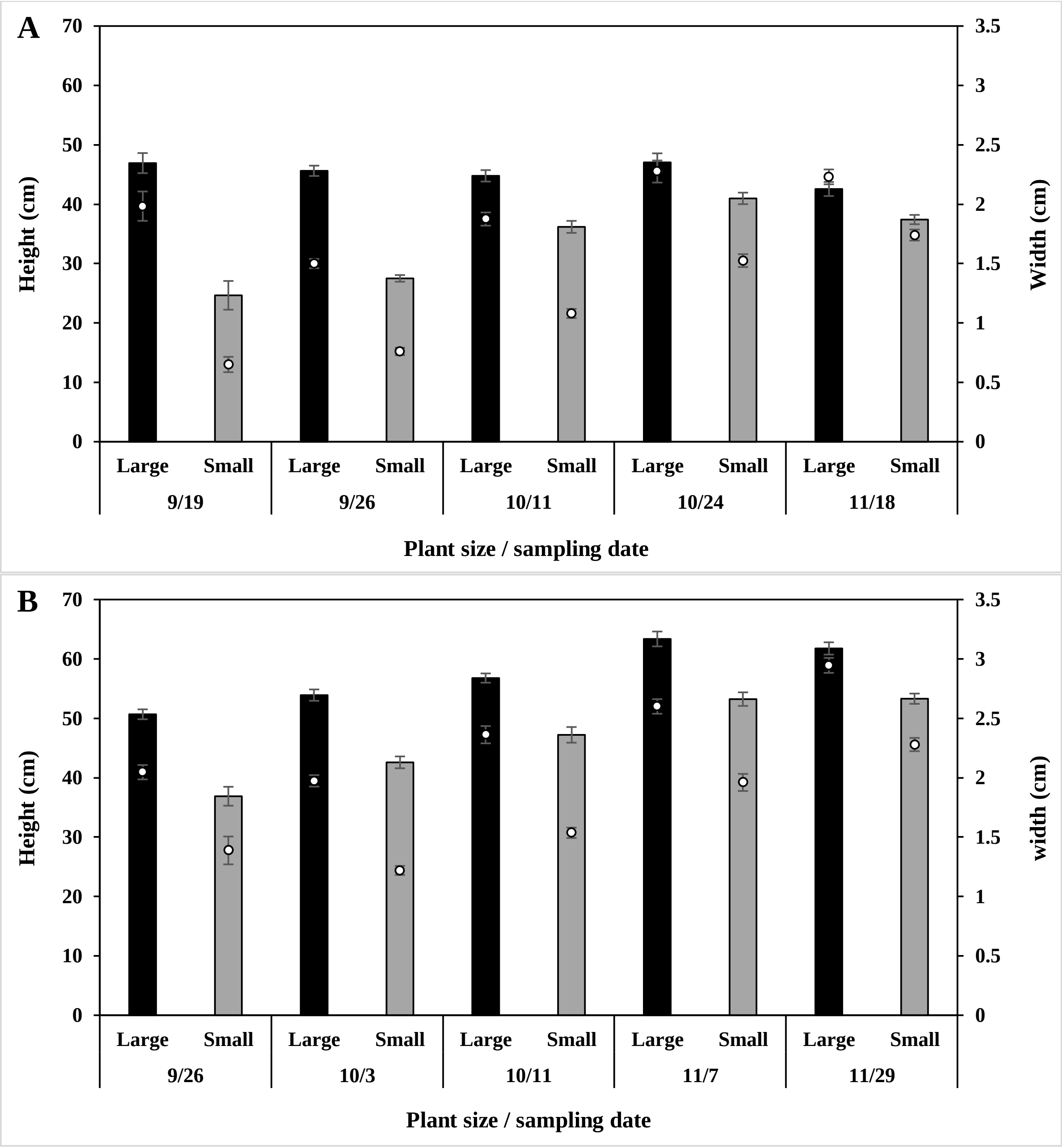

Supplementary Figure 2. Mean (± SE) height in bars and mean (± SE) width of the base of the leaves in dots of large and small scallions in (A) Hurley, NY and (B) Fenner, NY in 2022.

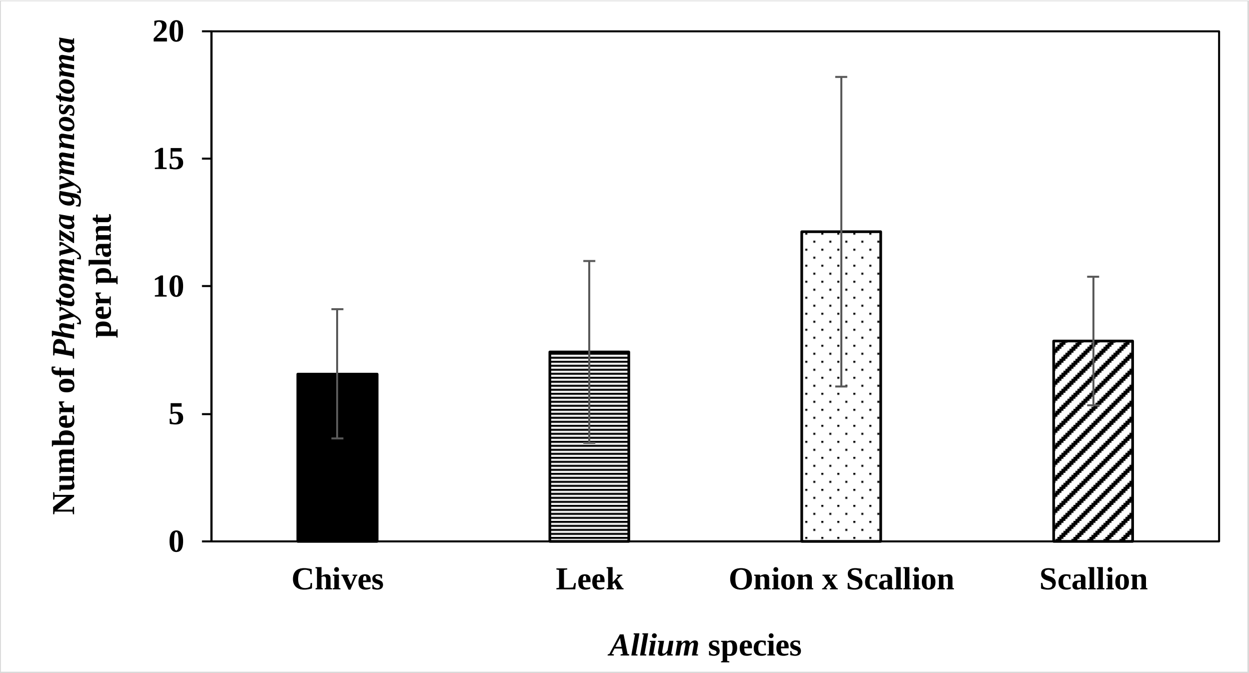

Supplementary Figure 3. Mean (± SE) number of *Phytomyza gymnostoma* (total number of eggs, larvae, and pupae) per plant at 21 days after flies were released in controlled environment choice tests. Least squares means of number of *P. gymnostoma* was not statistically different among *Allium* species (Tukey-HSD; P > 0.05; n = 7).

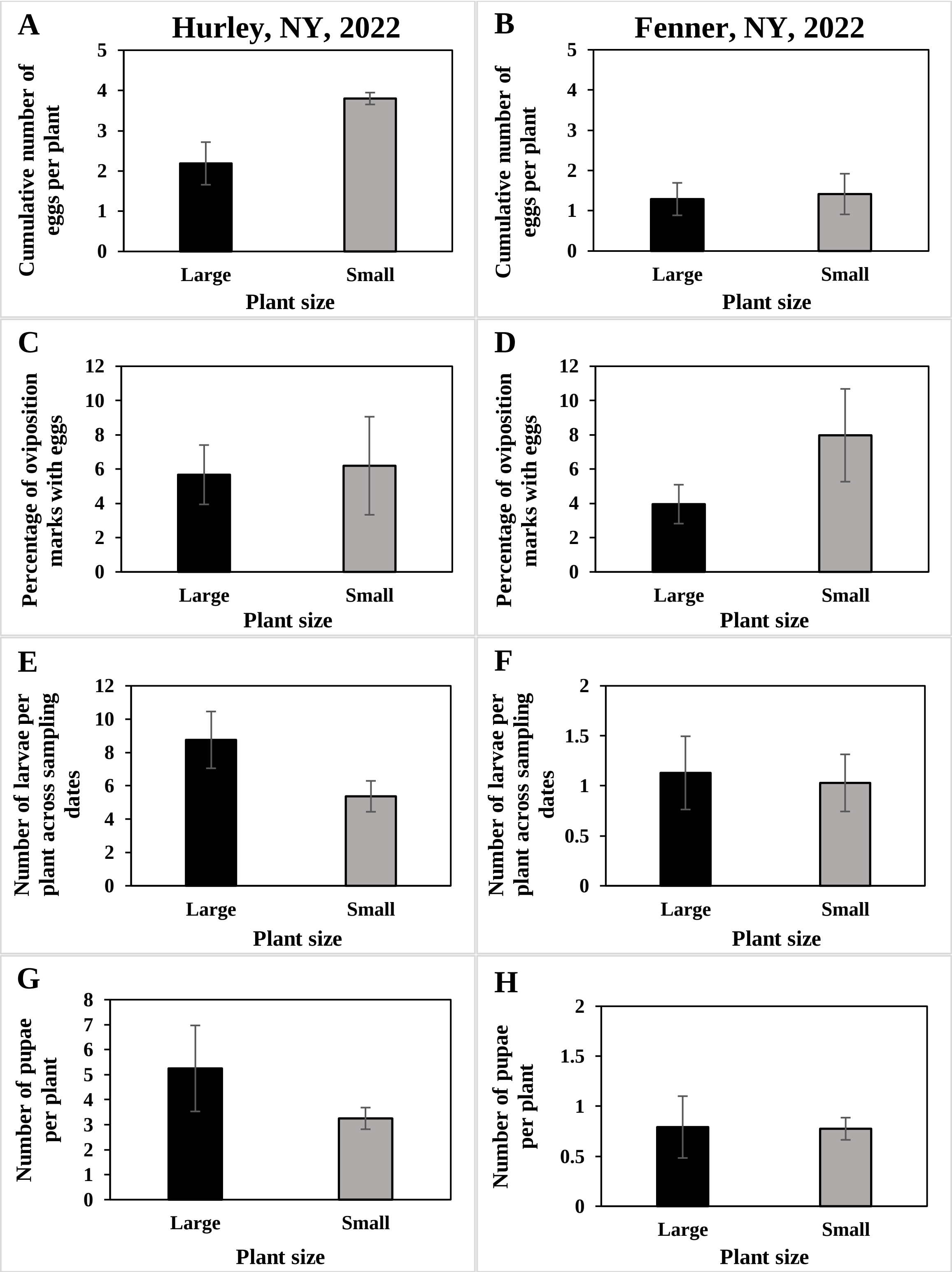

A
Hurley, NY, 2022
Cumulative number of eggs per plant
0
1
2
3
4
5
Large
Small
Plant size
B
Fenner, NY, 2022
Cumulative number of eggs per plant
0
1
2
3
4
5
Large
Small
Plant size
C
Percentage of oviposition marks with eggs
0
2
4
6
8
10
12
Large
Small
Plant size
D
Percentage of oviposition marks with eggs
0
2
4
6
8
10
12
Large
Small
Plant size
E
Number of larvae per plant across sampling dates
0
2
4
6
8
10
12
Large
Small
Plant size
F
Number of larvae per plant across sampling dates
0
0.5
1
1.5
2
Large
Small
Plant size
G
Number of pupae per plant
0
1
2
3
4
5
6
7
8
Large
Small
Plant size
H
Number of pupae per plant
0
0.5
1
1.5
2
Large
Small
Plant size

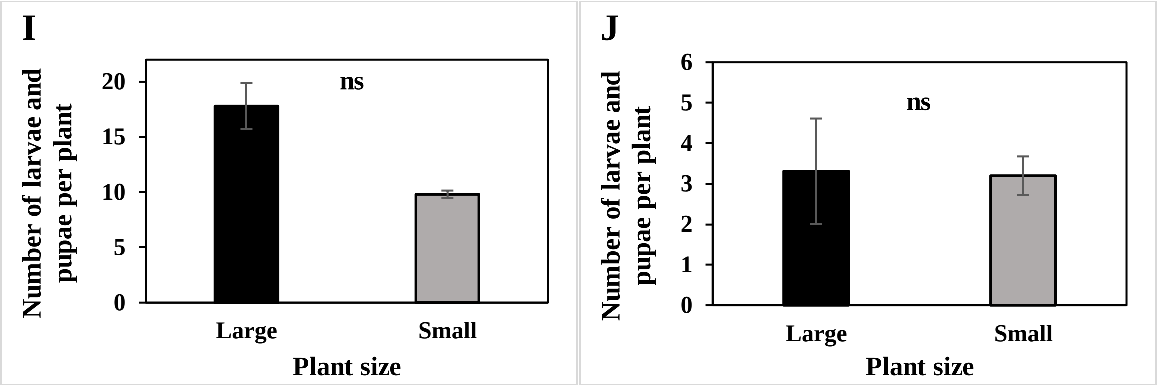

Supplementary Figure 4. Mean (± SE) cumulative number of *P. gymnostoma* eggs per plant in (**A)** Hurley, NY and **(B)** Fenner, NY in 2022. Mean (± SE) percentage of *Phytomyza gymnostoma* oviposition marks with eggs on the sampling date with the highest overall number during the season in **(C)** Hurely, NY on 26 september 2022 and **(D)** Fenner, NY on 3 October 2022. Mean (± SE) number of *P. gymnostoma* larvae per plant across sampling dates in (**E)** Hurley, NY and **(F)** Fenner, NY in 2022. Mean (± SE) number of *Phytomyza gymnostoma* pupae per plant on the last sampling date of the season in **(G)** Hurely, NY on 18 November 2022 and **(H)** Fenner, NY on 29 November 2022. Mean (± SE) number of *P. gymnostoma* larvae and pupae per plant on the sampling date with the highest overall number in **(I)** Hurely, NY on 24 October 2022 and **(J)** Fenner, NY on 07 November 2022. Note that the y-axis scales differ among parameters and locations. No significant differences in least squares means of number of *P. gymnostoma* (all parameters) between large and small scallions (Tukey-HSD; $P > 0.05$; $n = 4$).
